# A case report of a rare and challenging gangrenous splenic flexure volvulus in a pregnant patient

**DOI:** 10.1016/j.ijscr.2018.10.075

**Published:** 2018-11-02

**Authors:** Alison Bullen, Joel Lewin, Trent Cross, Benjamin Woolven

**Affiliations:** a University of Queensland, Brisbane, QLD, Australia; b James Cook University, Cairns Base Hospital, QLD, Australia

**Keywords:** Case report, Colonic volvulus, Pregnancy, Splenic flexure volvulus

## Abstract

**Introduction:**

Splenic flexure volvulus is a rare cause of intestinal obstruction and is uncommonly encountered in young patients from developed countries.

**Presentation of case:**

This report details a challenging case of a gangrenous large bowel volvulus in a 25 year old woman 20 weeks pregnant, highlighting the diagnostic and operative challenges that this rare clinical situation presents. After urgent MRI confirming a splenic flexure volvulus, an emergency open left hemicolectomy and end colostomy was performed. Following uneventful recovery and successful delivery of her baby at full term, she was reversed 6 months later.

**Discussion:**

Splenic flexure volvulus is a rare cause of large bowel obstruction, and as such can result in a delay in diagnosis, particularly in the setting of pregnancy. In this case, the patient was not diagnosed until day 3 of her admission when she decompensated with a rising white cell count.

**Conclusion:**

Splenic flexure volvulus is a rare occurrence. This case report of a young pregnant woman presenting with a large bowel obstruction due to splenic flexure volvulus highlights the importance of considering and investigating for this diagnosis.

## Highlights

• Splenic flexure volvulus is a rare cause of large bowel obstruction in pregnancy.
• We present a rare case of splenic flexure colonic volvulus in a 20 week pregnant young woman.
• Splenic flexure volvulus should be considered in pregnant patients with obstructive symptoms to enable early intervention.

## 1 Introduction

Colonic volvulus is an extremely rare, but potentially catastrophic, cause of intestinal obstruction in pregnant women. In 1953 Glazer and Adlersberg reported the first case of splenic flexure volvulus. In 2008 there were less than 100 reported cases in the scientific literature [1]. A volvulus results when a gas-filled loop of the colon rotates on its mesenteric axis greater then 180-degrees, causing obstruction of the intestinal lumen and mesenteric vessels [1]. This most commonly occurs at the sigmoid colon and caecum, however can also rarely occur at the splenic flexure, small bowel, transverse colon, stomach or gallbladder [2,3]. A splenic flexure volvulus is considered exceptionally rare, accounting for only 2% of colonic volvulus [4,5]. The following case is reported in line with the SCARE criteria case report guidelines [6].

## 2 Case report

We present the case of a 25 year old lady from Papua New Guinea who was admitted to the Cairns base hospital (CBH) with a 3 day history of abdominal pain and distension on a background of being 20 weeks pregnant. She had a slightly elevated white cell count upon admission, was haemodynamically stable and tolerating oral diet. The patient had last passed a bowel motion 2 days prior.

**Fig. 1:**
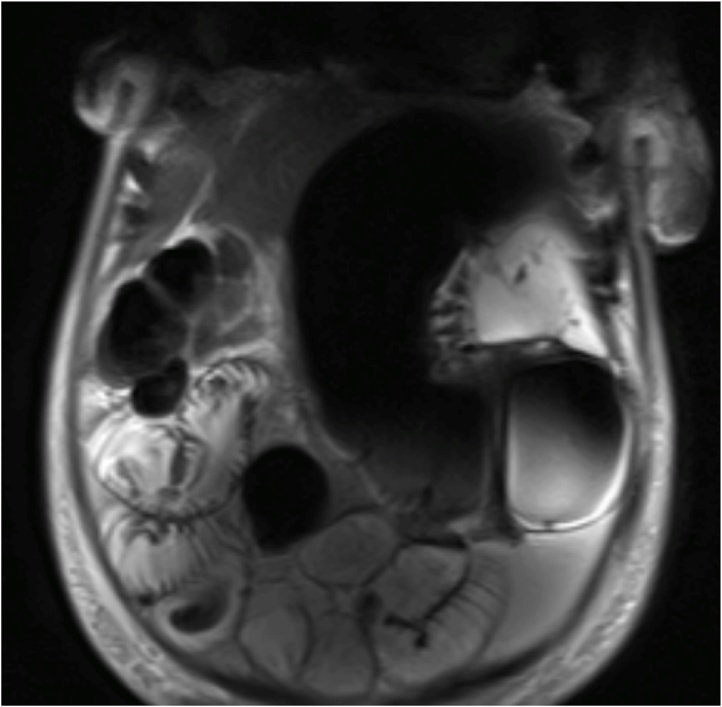
MRI abdomen, coronal view showing large bowel volvulus in the left upper quadrant.

**Fig. 2:**
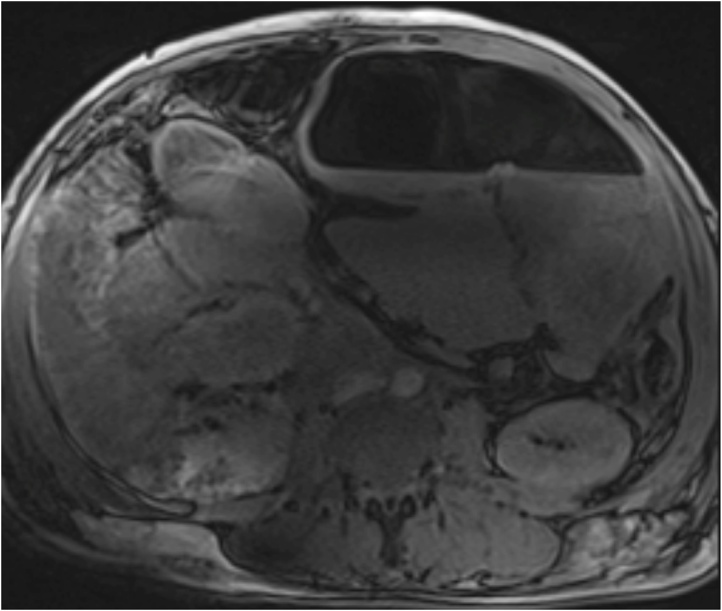
MRI abdomen, axial view showing large bowel volvulus in the left upper quadrant.

Plain abdominal X-ray and chest X-ray showed mild gaseous distension of her small bowel and right colon. Her admission diagnosis was possible abdominal tuberculosis (TB) given she had previous TB and was from an endemic area. On day 3 of her admission she became tachycardic and tachypnoeic with a grossly distended abdomen and worsening abdominal pain. She was febrile with a white cell count of 19 × 10^9^/L cells, lactate 3.2 mmol/L and haemoglobin of 126 g/L. An urgent MRI of her abdomen was performed, showing a likely large bowel volvulus with associated free fluid, but no perforation (Fig. 1, Fig. 2).

**Fig. 3:**
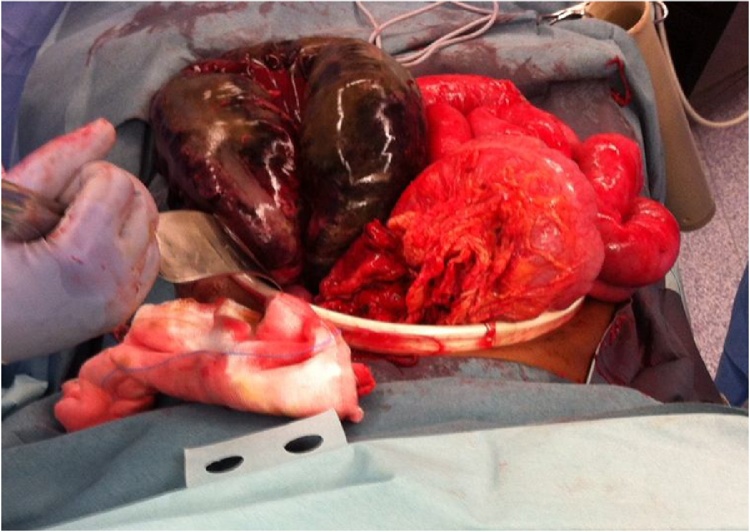
Intra-operative photo (1) of gangrenous splenic flexure.

**Fig. 4:**
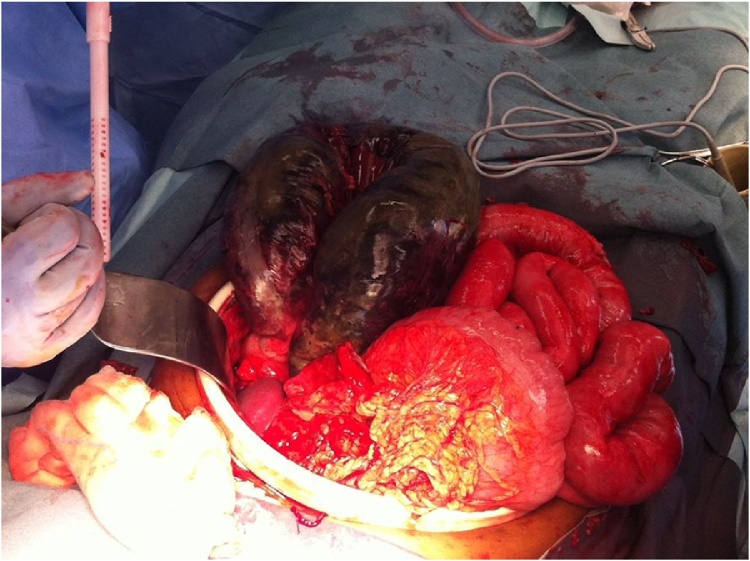
Intra-operative photo (2) of gangrenous splenic flexure.

Given the patients worsening clinical condition and imaging findings she was taken for an urgent laparotomy. A splenic flexure volvulus with gangrenous colon was found (Fig. 3, Fig. 4) and the patient underwent a left hemicolectomy and end colostomy. The decision was made not to perform a primary anastomosis given her clinical condition and the risk presented by a potential anastomotic leak adjacent to the gravid uterus. She had pre and postoperative obstetric team review. The patient recovered well and delivered a healthy baby at full term 4 months later. She underwent a reversal of her colostomy 6 months later and was discharged home without incident. Happily both mother and baby were both well at follow up in the outpatient clinic.

## 3 Discussion

The uncommon occurrence of splenic flexure volvulus can be attributed to the splenic flexure being tethered by 3 ligamentous attachments, making it less likely to malrotate [7]. The underlying cause of this condition is yet to be precisely defined, however it is widely hypothesised that prior abdominal surgery, chronic constipation and colonic dysmotility predispose an individual to a colonic volvulus [3,5,[7], [8], [9], [10]]. An increased risk of volvulus has been associated with Crohn’s disease, Chagas disease and pregnancy [3]. In developed countries this condition is often described in an elderly population, with a mean age of onset 70 [2,3], making it an uncommon cause for an acute abdomen in a young patient.

Ballantyne et al (1985) described a single centre series of 137 patients with colonic volvulus over a 20 year period. They described 52% of these as caecal in origin, 43% sigmoid, 3% transverse colon, and only 2% as splenic flexure. [9] Pregnancy is thought to increase the risk of sigmoid volvulus as the enlarging uterus may cause a redundant sigmoid colon to be pushed out of the pelvis and twist on its mesentery, and is more commonly seen in multiparous women in their 3^rd^ trimester. [11,12] A 2014 review of the literature identified less than 90 reported cases of sigmoid volvulus during pregnancy [13]. Volvulus of the splenic flexure is less common due to its relative immobility, being held in position by the phrenicocolic, gastrocolic, and splenocolic ligaments.

In this case there was a delay in the diagnosis due to atypical presentation in an initially stable pregnant women. This delay was due to attributing her symptoms to other pregnancy related conditions, hesitation to use imaging such as X-ray and CT, the rare occurrence of the condition and the patient’s previous diagnosis of TB. MRI was performed due to pregnancy. MRI is not the gold standard for imaging diagnosis of a colonic volvulus, however in situations of a pregnant patient, it should be used to avoid radiation exposure to the foetus.

CT scan is typically used for diagnosis, showing the characteristic appearance of markedly dilated, air-filled colon with an abrupt termination at the anatomic splenic flexure [4]. In the absence of CT scan availability an abdominal X-ray can be performed. The surgery, although not particularly challenging from a technical operative standpoint, does provide difficult intraoperative decisions. Options include primary anastomosis with or without a protective ileostomy, or colectomy and end colostomy. In the acutely unwell patient, resection and an end colostomy is generally the safest option for the patient, however in the situation of a splenic flexure volvulus, a transverse colon stoma will be required, making subsequent reversal more technically difficult. Reversal can be undertaken in the elective setting by an experienced colorectal surgeon usually 3–6 months after the initial surgery. Immediate surgery in patients with signs or symptoms of peritonitis is indicated and the diagnostic dilemma presented in a patient such as this should not delay surgery.

## 4 Conclusion

Volvulus of the splenic flexure is a rare cause of large-bowel obstruction, with predisposing factors including the congenital absence, or surgical excision, of gastrocolic, phrenocolic and splenocolic ligament and the presence of a long mesentery. When these elements are present, the splenic flexure will have higher mobility and risk of volvulus. Splenic flexure volvulus is a rare occurrence in pregnant patients, however this case demonstrates that it should be considered in the differential diagnosis for a patient presenting with acute abdominal pain. Early imaging with MRI in the case of pregnancy is indicated and early surgical intervention once the diagnosis is established.

## Conflict of interest

There are no conflicts of interest, financial, personal or otherwise which could influence bias.

## Funding source

No funding was needed for this case report, except for the amount required if published.

## Ethical approval

Ethical approval was not required for this case.

## Consent

Consent was obtained from the patient to write this case report using de-identified information and de-identified accompanying radiological and clinical images.

## Author contribution

Dr Alison Bullen – Corresponding author. Review of patient notes, writing of manuscript, and critically analysed and approved final submission.

Dr Joel Lewin – Writing of manuscript, editing and approved final submission.

Dr Trent Cross – Critical analysis and revision of draft, approved final submission.

Dr Benjamin Woolven – Critical revision of draft and approved final submission.

## Registration of research studies

N/A.

## Guarantor

Dr Trent Cross – General Surgery Registrar, Cairns Hospital, Cairns, Queensland

## Provenance and peer review

Not commissioned, externally peer reviewed.
